# Radicular compression syndrome after exercise in a young patient: not everything is a herniated disk!

**DOI:** 10.1590/0100-3984.2017.0080

**Published:** 2018

**Authors:** Eduardo Pontes Reis, Nivaldo Adolfo Silva Junior, Simone Appenzeller, Fabiano Reis

**Affiliations:** 1 Universidade Estadual de Campinas (Unicamp), Campinas, SP, Brazil.

**Figure 1:**
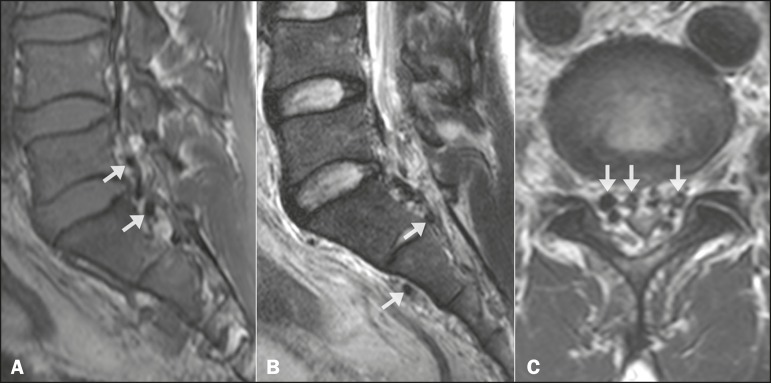
Magnetic resonance imaging, including a sagittal T1-weighted image (**A**), a sagittal T2-weighted image (**B**) and an axial T2-weighted image (**C**), showing enlargement of the epidural veins with compression of lumbar roots (arrows).

**Figure 2:**
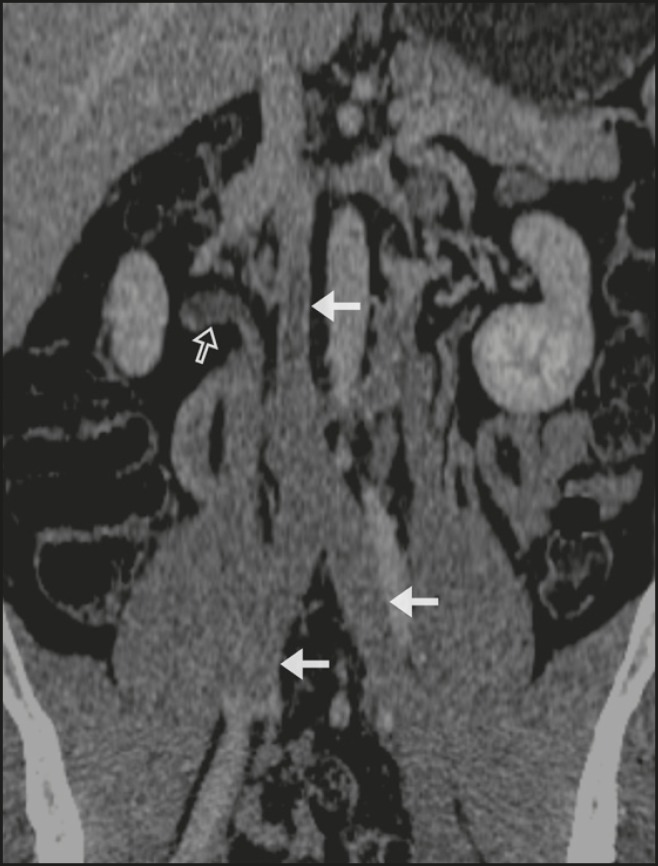
Abdominal coronal reformatted computed tomography scan showing extensive thrombosis of the IVC, common iliac veins (arrows), left external iliac vein, and right accessory renal vein (open arrow).

Dear Editor,

A 34-year-old previously healthy man presented with a complaint of sudden-onset, progressive low back pain radiating to the lower limbs after running. The symptoms had begun three weeks earlier, with acute worsening during the last four days. The physical examination was normal except for mild lower left limb edema. A lumbosacral magnetic resonance imaging scan showed dilated vessels in the epidural space, intervertebral foramen, and anterior paraspinal space (Figure 1). Complementary abdominal computed tomography angiography of the abdomen showed thrombosis of the inferior vena cava (IVC), common iliac veins, left external iliac vein, and right accessory renal vein (Figure 2). The patient was submitted to mechanical thrombectomy, followed by chemical thrombolysis and long-term anticoagulation therapy, with resolution of symptoms. A control computed tomography scan, obtained six months later, showed patency of the IVC and common iliac veins, together with chronic thrombosis of left internal iliac vein. Further investigations for acquired thrombophilia were negative.

Engorgement of the epidural venous plexus secondary to IVC thrombosis is a rare cause of lower back pain by compression of nerve roots, and it is a differential diagnosis to disc-degenerative changes^(^^1^^,^^2^^)^. Paksoy et al.^(^^3^^)^ identified IVC obstruction as a cause of radiculopathy in 0.13% patients who underwent magnetic resonance imaging for the investigation of radicular symptoms mimicking lumbar disc herniation or spinal stenosis; in all of those patients, it was the first episode (of low back pain).

The vertebral venous system consists of the internal vertebral veins or epidural veins (internal network) and the lumbar segmental veins or paravertebral veins (external network), which communicate with the common iliac veins, azygous system, and IVC, through the lumbar segmental veins^(^^1^^,^^3^^)^. Although it normally presents flow that is ascendant and away from the spinal flow, it can present retrograde flow because of its valveless nature, becoming an alternative pathway between the iliac-caval and azygous systems^(^^3^^)^. The internal and external networks communicate through the radicular veins, which have an intimate relationship with the nerve roots and, when engorged, may mimic radicular compression^(^^1^^-^^4^^)^. Possible causes of engorgement of the epidural veins of the back include vascular malformations of the epidural venous plexus; IVC thrombosis, related to pregnancy or not^(^^5^^-^^8^^)^; portal hypertension^(^^2^^)^; spinal dysraphism^(^^3^^)^; epidural lipomatosis^(^^3^^)^; Budd-Chiari syndrome^(^^4^^,^^9^^)^; and intracranial hypotension^(^^2^^)^.

Engorgement of epidural vessels should immediately raise the suspicion of cava thrombosis, and the radiologist plays an important role in correlating it with the possible underlying etiologies. Due to its severity, deep venous thrombosis, as seen in this case, must be promptly diagnosed and treated.
